# Fatty acid and retinol-binding protein: A novel antigen for immunodiagnosis of human strongyloidiasis

**DOI:** 10.1371/journal.pone.0218895

**Published:** 2019-07-22

**Authors:** Leila Masoori, Ahmad Reza Meamar, Mojgan Bandehpour, Andrew Hemphill, Elham Razmjou, Kobra Mokhtarian, Mona Roozbehani, Alireza Badirzadeh, Nahid Jalallou, Lame Akhlaghi, Reza Falak

**Affiliations:** 1 Department of Parasitology and Mycology, School of Medicine, Iran University of Medical Sciences, Tehran, Iran; 2 Department of Biotechnology, School of Advanced Technologies in Medicine, Shahid Beheshti University of Medical Sciences, Tehran, Iran; 3 Cellular and Molecular Biology Research Center, Shahid Beheshti University of Medical Sciences, Tehran, Iran; 4 Institute of Parasitology, Vetsuisse Faculty, University of Bern, Bern, Switzerland; 5 Clinical Biochemistry Research Center, Basic Health Sciences Institute, Shahrekord University of Medical Sciences, Shahrekord, Iran; 6 Department of Medical Laboratory Sciences, Faculty of Allied Medicine, AJA University of Medical Sciences, Tehran, Iran; 7 Immunology Research center, Iran University of Medical Science, Tehran, Iran; 8 Department of Immunology, School of Medicine, Iran University of Medical Sciences, Tehran, Iran; Taipei Medical University/Medicine, TAIWAN

## Abstract

The tenacious human parasitic helminth *Strongyloides stercoralis* is a significant health problem worldwide. The current lack of a definitive diagnostic laboratory test to rule out this infection necessitates designing more specific diagnostic methods. Fatty acid and retinol-binding protein (FAR) plays a crucial role in the development and reproduction of nematodes. We generated a recombinant form of this protein and determined its applicability for immunodiagnosis of *S*. *stercoralis*. The L3 form of *S*. *stercoralis* was harvested and used for RNA extraction and cDNA synthesis. The coding sequence of *S*. *stercoralis* FAR (SsFAR) was cloned into pET28a(+) vector, expressed in *E*. *coli* BL21 and purified. ELISA and immunoblotting were employed to determine the specificity and sensitivity of rSsFAR using a set of defined sera. In addition, we analyzed the phylogenetic relationship of SsFAR with different FAR sequences from other nematodes. The cloned SsFAR had an open reading frame of 447 bp encoding 147 amino acids, with a deduced molecular mass of 19 kD. The SsFAR amino acid sequence was 93% identical to FAR of *S*. *ratti*. For differential immunodiagnosis of strongyloidiasis, rSsFAR exhibited 100% sensitivity and 97% specificity. However, cross-reactivity with FAR proteins of other parasites, namely *Toxocara canis* and *Echinococcus granulosus*, was noted. Our results provide a novel approach for immunodiagnosis of *S*. *stercoralis* infections using rSsFAR with reliable sensitivity and specificity.

## Introduction

Strongyloidiasis, caused by the tenacious human parasitic helminth *Strongyloides stercoralis*, is a significant health problem [1, 2]. The precise prevalence of strongyloidiasis is not clear, but it is estimated to affect at least 370 million people worldwide [3]. Strongyloidiasis is endemic in some part of Iran but depending on the diagnosis methods, different prevalence rates were reported ranging from 0.03 to 42% [4–9].

This soil-transmitted nematode enters the host through exposed skin and consequently migrates to the small intestine [10]. Among helminths affecting humans, *S*. *stercoralis* has a unique property of auto-infection and is capable of propagating within the host [11–13]. Strongyloidiasis may remain undiagnosed and untreated for decades, and upon immune-suppression can lead to hyperinfection, severe morbidity and mortality [14].

The detection rate of the infective L3 larval form (iL3) of *S*. *stercoralis* by conventional methods is low. To achieve a higher sensitivity, examination of multiple samples is required [15, 16]. Up to now, the definitive diagnosis of strongyloidiasis has been achieved by coprological procedures. However, conventional microscopic methods may fail to determine the infection, because in approximately 70% of the cases the parasite load is below the detection limit and the larval output by the adult worms is irregular [2, 3, 17–19].

In recent years, immunodiagnostic methods, particularly enzyme-linked immuno-sorbent assay (ELISA), were improved and are now regarded as the method of choice for evaluating a number of different helminthiasis [3, 20–22]. For instance, the high immunogenicity of cathepsins, which are found in large quantities in some helminths excretory and secretory (E/S) products [23, 24], resulted in the development of specific diagnostic methods. In addition, some of these antigens were also exploited as promising vaccine candidates [25–28]. However, a major caveat for serological approaches in diagnosis of helminthiasis is the cross-reactivity with several other soil-transmitted nematodes, including hookworm and *Trichostrongylus* species [29, 30]. Consequently, diagnosis of strongyloidiasis requires more sensitive techniques, especially in those patients who suffer from mild infections with scarce excretion of larvae and also in immunocompromised individuals [17]. Therefore, identification, cloning and characterization of novel recombinant immunogens, with reliable sensitivity and specificity, could simplify some issues of the immunodiagnosis in this field.

Usually, proteins which are applied in immunodiagnostic studies are abundantly expressed and could be easily purified. In addition, high immunogenicity could potentially be translated into protective immunity. Several small proteins which demonstrate the mentioned characteristics, including fatty acid binding (FAB) and fatty acid and retinol-binding (FAR) proteins are expressed at the surface of nematodes and are believed to be critical for helminth survival [31, 32]. Fatty acids are essential for lipid biosynthesis, neurological processes and cuticle assembly of parasites [33, 34]. Moreover, it was previously observed that FAB is abundant in the fluid surrounding the embryo in eggs and might be essential in nutrient acquisition and maintenance of the eggshell of the developing larvae; thus FAB is regarded as a crucial molecule in larva embryogenesis [35]. FAR also participates in intracellular and intercellular lipid signaling and plays role in host defense mechanisms and pathogenesis of nematodes [25, 36–42]. Moreover, FAR has been shown to be suitable for immunodiagnosis and has confirmed good potency in experimental vaccinology [25, 43]. Basavaraju et al. showed that *Ancylostoma caninum* FAR might enhance the infectivity of the parasite by reducing the available retinol molecules that would be necessary for repairing damaged tissues during hookworm attachment [36]. The 14-kDa FAB protein of *Schistosoma mansoni* (Sm14) has been proposed as a vaccine candidate against *S*. *mansoni* infections in human and *Fasciola hepatica* against cattle and sheep [44, 45].

Characterization of the FAB or FAR proteins of *S*. *stercoralis* could help in understanding their role in the pathogenesis of the infective form of this parasite. In this study, we generated a recombinant version of FAR protein from the L3 stage of *S*. *stercoralis* (rSsFAR) and determined its immunogenicity and usefulness in the immunodiagnosis of strongyloidiasis.

## Materials and methods

### Ethics statement

The applied research protocols were approved by the Ethics Committee of Iran University of Medical Sciences (code number IR.IUMS.REC 1395–9221577205). All participants were adult and informed consent was obtained from each patient or control before participating in the study or sample donation. *Strongyloides stercoralis* infected patients’ serum and stool samples were collected from endemic areas including Mazandaran, Guilan and Khuzestan provinces of Iran and anonymized before including in the study.

### Isolation of parasites

Stool samples from microscopically confirmed *S*. *stercoralis* infected patients were cultivated on nutrient agar plates at 25°C. After three to four days of incubation, L3 larvae were observed on the surface of the plates. After microscopic examination and observation of the active movement and consequently the survival of the filariform larvae, the larvae were harvested and washed several times with ice-cold phosphate buffered saline (PBS) to decrease bacterial contamination [46].

### Serum samples

A total of 113 serum samples were collected and categorized into one of following groups: group A included 33 serum samples from individuals who were diagnosed with obvious *S*. *stercoralis* infection by stool examination. These samples were regarded as positive and used for detection of specific antibodies; group B was composed of 40 serum samples from individuals who were diagnosed as having other parasitic disease including hydatidosis (N = 14), toxocariasis (N = 9), fasciolosis (N = 5), visceral leishmaniasis (N = 8), giardiasis (N = 3) and trichostrongyloidiasis (N = 1). These samples were applied to assess serological cross-reactivity. Group C included 40 sera from individuals with no history of parasitic disease. These samples were regarded as negative controls.

### Cloning, expression and purification of rSsFAR

Total RNA was extracted from the iL3 form of *S*. *stercoralis* using Trizol reagent (Invitrogen, Carlsbad, CA, USA), according to the manufacturer’s instructions with some modifications. In brief, Trizol was added to the parasite pellet and the mixture was frozen and thawed several times, and ground with a pestle and mortar to crush the larvae. The homogenate was centrifuged at 13,000 x *g* for 10 min and the supernatant was collected and used for RNA extraction. The quality and quantity of the extracted RNA was evaluated by agarose gel electrophoresis and Nanodrop spectrophotometry (Thermo Scientific, USA), respectively. Complementary DNA (cDNA) synthesis was done using oligo (dT) primers using a commercial kit (Biofact, South Korea) and the quality of the cDNA was checked by beta actin specific primers.

Since the nucleotide sequence of *S*. *stercoralis* FAR was unknown, we used the *S*. *ratti* FAR sequence (GenBank accession number of CEF68179.1) for primer design through aligning the coding sequence of *S*. *ratti* FAR with other available FAR sequences. The cDNA encoding the full-length sequence of SsFAR was amplified by PCR, using specific forward primer (5ˈ ATA GGA TCC TCA GAT GAC TTA TTA GAA TCT 3ˈ) and reverse primer (5ˈ ATA CTC GAG AAG TGG TAC CGG AAG GTG 3ˈ). Notably, the underlined sequences indicate *BamHI* and *XhoI* restriction enzyme cutting sites, respectively. The PCR product was cloned into the pTG19-T/A vector (Vivantis, Malaysia) and sequenced. White colonies harboring the target construct was selected for enzymatic digestion. The vector was digested with *BamHI* and *XhoI* (Jena Bioscience, Jena, Germany) and the insert was ligated into the pET28a(+) expression vector (Novagen, USA), and was used for transformation of *E*. *coli* DH5α. The desired plasmid was purified by a commercial plasmid purification kit (Yekta Tajhiz Azma, Tehran, Iran) and transformed into *E*. *coli* BL21(DE3) for optimal expression of recombinant sSFAR (rSsFAR). The bacterial cells were grown in LB medium containing 50 μg/ml kanamycin (Biosynth AG, Switzerland) at 37°C to reach OD_600_ = 0.6. Finally, recombinant protein expression was triggered by addition of 1 mM isopropyl-thiogalactoside (IPTG, Sigma, USA) and incubation of the bacterial suspension for 4–6 hours at 37°C. Subsequently, the bacterial cells were harvested by centrifugation and following cell lysis, rSs FAR was purified using a Hi-Trap chelating Sepharose column (Ni-NTA agarose, Qiagen, Hilden, Germany) [47].

### SDS-PAGE and Western blotting

The quality of the purified rSsFAR was assessed by SDS-PAGE using 15% polyacrylamide gels, followed by transfer to polyvinylidene difluoride (PVDF) membranes and immunoblotting with anti-His-tagged antibody (Sigma-Aldrich, Saint Louis, USA) or with patients' sera. In brief, the transfer was done by semidry blotting (PeQLab, Belgium), and the membranes were cut into strips and blocked with 5% skimmed milk in PBS overnight at 4°C by constant shaking on a rocker. After washing with PBS containing 0.05% Tween 20 (PBST), strips were incubated with horseradish peroxidase (HRP)-conjugated anti-His-tag antibody (1:2000 dilution in BSA 1%) or with patients’ sera diluted 1:100 in 1% BSA, for 2 h at room temperature (RT). Subsequently, the membranes were washed three times, for a total of 15 min in PBST. The anti-His-tag-reactive bands were visualized by diaminobenzidine (DAB) substrate (Roche, USA). Strips that were incubated with the patients’ sera were extensively washed and incubated with HRP-conjugated sheep anti-human IgG (Sina Biotech, Iran, 1:2000 dilution) for 1 h at RT. Finally, the immunoreactivity of the specific antibodies in patient samples were visualized as described above.

### Optimization of SsFAR-specific ELISA

The titer of the specific antibody in patient sera against the purified protein was analyzed by an indirect ELISA developed in-house. The 96-well microtiter plates (Nunc, Denmark) were coated overnight at 4°C with 2.5 μg/ml of rSsFAR solubilized in 0.2 M bicarbonate buffer pH 9.6. Subsequently, the wells were washed three times with PBST and blocked with 2% BSA/PBST (2h in 37°C). Patient sera (1:100 diluted) were added 100 μl/well and the plates incubated for 2 hours at 37°C. After four washes with PBST, 100 μl of HRP-conjugated sheep anti-human antibody, diluted 1:1000, were added to the wells (Sina Biotech, Iran). Following 1 h incubation at 37°C the wells were washed four times with PBST and then 100 μl of tetramethyl-benzidine (TMB) substrate was dispensed into each well. The reaction was stopped after 15 min by addition of 100 μl of 2N H_2_SO_4_ and the optical density (OD) was read by an ELISA reader (BioHit, Helsinki, Finland) at 450 nm versus 630 nm as reference filter.

### DNA sequence analysis

The SsFAR-1 cDNA and deduced amino acid sequence was aligned with sequences from FAR homologues of other helminths retrieved from GenBank, including *S*. *ratti*, *Haemonchus contortus*, *Caenorhabditis elegans*, *C*. *briggsae*, *Onchocerca volvulus*, *Wucheria*. *bancrofti*, *Brugiya malayi*, *B*. *pahangi* and *Toxocara canis* using online ClustalW (http://www.clustal.org/clustal2/) and Multalin (http://multalin.toulouse.inra.fr/multalin/) softwares (S1 and S2 Files). Overlapping contigs were edited at each consensus position through Sequencher Tmv.4.1.4 Software (Gene Codes Corporation). The distance between all sequences was calculated and phylogenetic trees were constructed by maximum-likelihood in the Kimura two-parameter model by the MEGA 5.05 software (Arizona State University, Tempe, USA).

### Statistical methods

All data were analyzed using GraphPad Prism version 6.0. (GraphPad Software, CA, USA). The optimum cut-off value for ELISA assay, with a 95% confidence interval (CI), was obtained by receiver-operating characteristic (ROC) curve analysis. ANOVA was used to detect significant differences between the experimental groups. *P* values lower than 0.05 were considered statistically significant.

## Results

Plate-agar cultivation of the feces from 33 patients with microscopically confirmed *S*. *stercoralis* infection showed that 28 patients suffered from hyper-infection, and this resulted in the propagation of numerous L3 forms of the parasite, which were collected and used for protein and RNA extraction.

Western blotting of the crude extract of the L3 form with the pooled sera from hyper-infected patients confirmed intensive antibody reactivity of a native protein with an apparent molecular weight of 16 kDa. Although, immunoblotting of the crude extract with individual patient sera showed different patterns of immunoreactivity, all strips demonstrated reactivity with a 16 kDa protein band.

**Fig 1:**
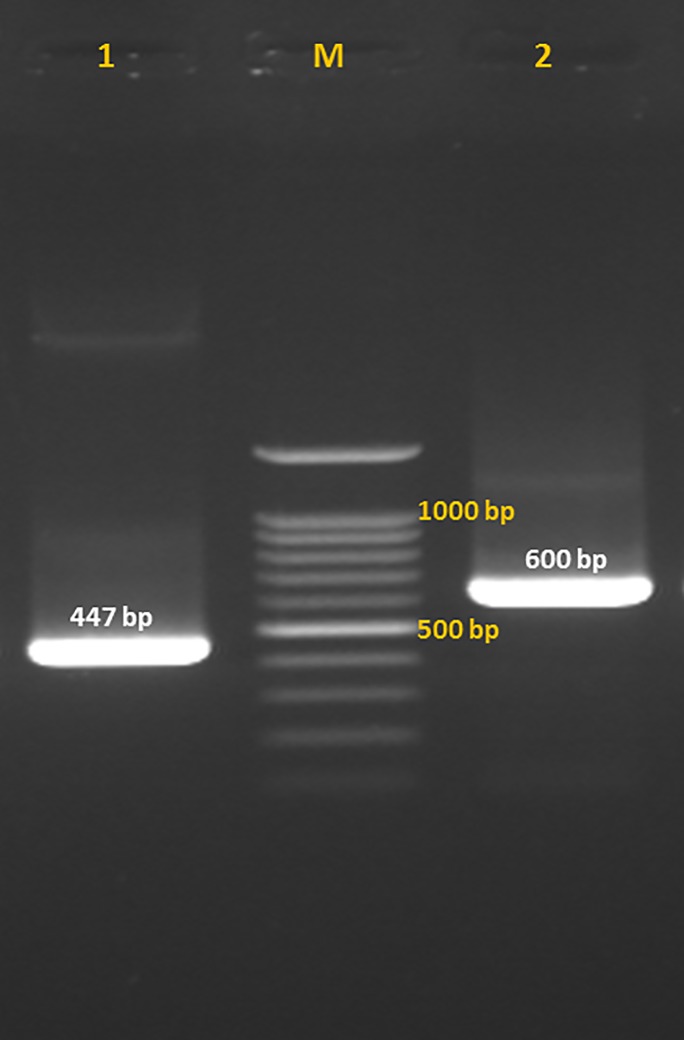
Fatty acid and retinol-binding protein (FAR) gene fragments of *Strongyloides stercoralis*. Fatty acid and retinol binding protein (FAR) sequence of infective larvae of *Strongyloides stercoralis* was amplified by conventional PCR and following insertion in pTG19-T/A vector was rechecked. Lane 1: PCR product of the amplification of FAR gene from the cDNA of infective larvae of *S*. *stercoralis*; Lane 2: PCR amplification of FAR gene which was inserted into pTG19-T/A vector with M13-specific primers: Lane M: 100 bp DNA ladder (Cinnagen, Tehran, Iran).

RNA was successfully extracted and used for cloning of SsFAR. PCR amplification of the target sequence yielded a single 447 bp band (Fig 1; PubMed Ac No: LC315684) which showed 92% identity with *S*. *ratti* FAR (PubMed Ac No: XM_024653954.1) as well as significant homologies to FAR sequences from other nematodes (S1 File).

**Fig 2:**
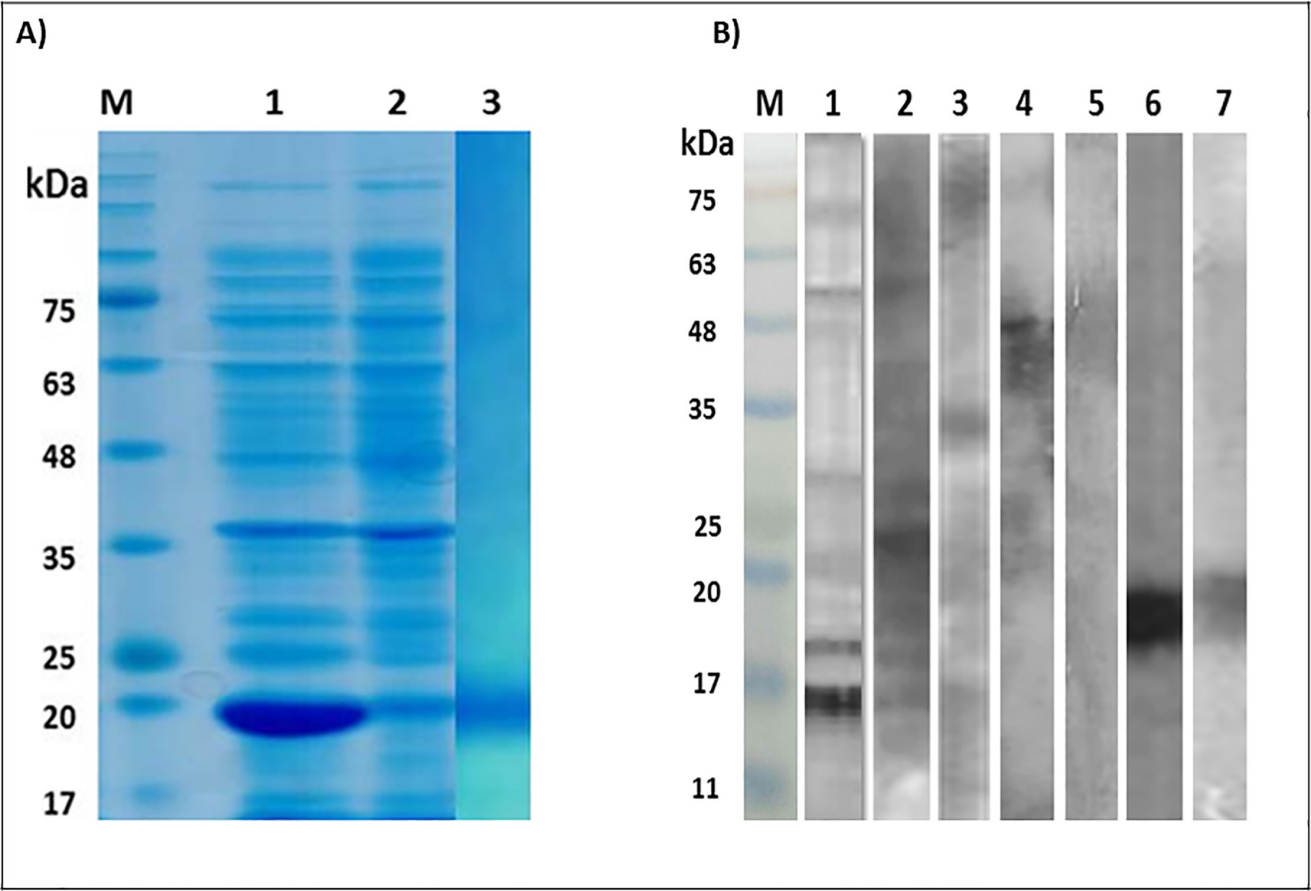
SDS-PAGE and immunoblotting of the crude extract, as well as recombinant fatty acid and retinol-binding protein of *Strongyloides stercoralis*. Fatty acid and retinol-binding protein (FAR) was expressed in a prokaryotic host and the immunoreactivity of the purified protein was analyzed by immunoblotting. **A:** *Strongyloides stercoralis* FAR gene was cloned in pET28a(+) plasmid, transformed in *E*. *coli* BL21 and the expression of the rFAR protein was induced by 1mM IPTG within four hours of incubation at 37°C. The bacterial cells were lysed and following centrifugation the contents of the precipitated and the supernatant fractions was analyzed by 15% SDS-PAGE gels for evaluation of the expression of the target protein. The slabs were stained with Coomassie brilliant blue G250. Lane M: Protein molecular weight marker. Lane 1: Supernatant of the lysed bacterial cells showing overexpression of a 19 kDa recombinant protein; Lane 2: Precipitate of the lysed bacterial cells, Lane 3: Purified rFAR. **B:** The crude extract from infective larvae of *S*. *stercoralis* and purified rFAR was immunoblotted with pooled sera from hyper-infected strongyloidiasis patients, as well as toxoariasis and hydatidosis patients. Furthermore, anti-his-tagged antibody was used for characterization of the recombinant protein. M: Protein molecular weight marker, Lane 1–2: Immunoblotting of the crude extract with pooled sera of five strongyloidiasis patients; 3: Immunoblotting of the crude extract with polled sera of two hyper-infected patients; Lane 4: Immunoblotting of the crude extract with toxocariasis patients’; Lane 5: Immunoblotting of the crude extract with pooled sera of hydatidosis patients; Lane 6: Immunoblotting of rFAR protein with anti-his-tagged antibody, Lane 7: Immunoblotting of rFAR protein with pooled sera of strongyloidiasis patients.

SDS-PAGE analysis of the purified rSsFAR revealed expression of a single 19 kDa protein band, which was consistent with the predicted size. The immunoreactivity of the recombinant protein was simultaneously confirmed by ELISA and Western blotting. By immunoblotting, the pooled sera of the strongyloidiasis patients showed strong immunoreactivity with the recombinant protein (Fig 2).

**Fig 3:**
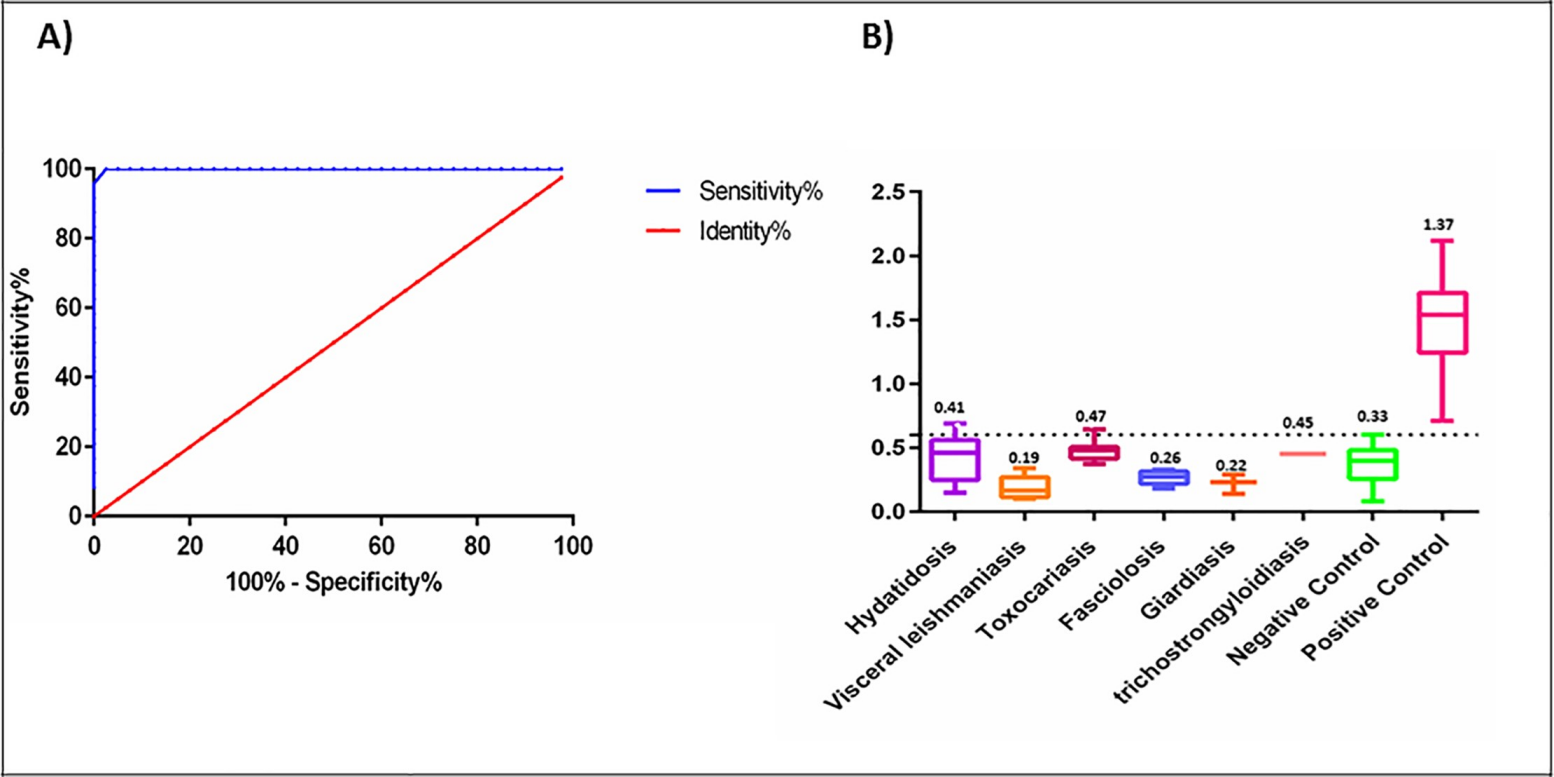
Receiver-operating characteristic (ROC) curves and the Cross-reactivity of rFAR sera from patients with other parasitic diseases. **A:** The ROC curve was plotted using optical density (OD) obtained in ELISA for 33 serum samples from *S*. *stercoralis*-infected patients and 40 healthy controls. The area under the curve (AUC) for recombinant Fatty acid and retinol-binding protein (rFAR) was 0.99. **B:** Cross-reactivity of rFAR with antibodies against other parasitic diseases was determined by indirect ELISA. Sera from some other parasitic diseases were examined with rFAR-coated ELISA plates and did not show considerable cross-reactivity.

ROC analysis of the ELISA results confirmed that the area under curve (AUC) value was 0.99 and the cut-off value was 0.6. All *S*. *stercoralis* infected patient sera showed a high frequency of reactivity with the expressed protein, resulting in a mean OD of the patients' sera that was significantly higher than controls (1.37±0.36 versus 0.33±0.12). The sensitivity and specificity of the reaction was determined to be 100% and 97%, respectively (Fig 3A and S3 File).

The expressed rSsFAR protein showed no significant cross-reactivity with sera obtained from healthy individuals; however, we observed immunoreactivity of this protein with sera of patients with other parasitic disease (Table 1, Fig 3B and S4 File).

**Table 1:** Cross-reactivity of the sera of the patients with various parasitic diseases with recombinant fatty acid and retinol-binding protein (rFAR) of *S*. *stercoralis*.

Infection	Tested sera (%)	Mean±SD	Positive IgG-ELISA No. (%)
**Hydatidosis**	14 (35%)	0.41 ±0.17	2 (14.2%)
**Toxocariasis**	9 (22.5%)	0.19 ±0.08	1 (11.1%)
**Fascioliasis**	5 (12.5%)	0.47±0.07	0
**Visceral leishmaniasis**	8 (32%)	0.26±0.05	0
**Giardiasis**	3 (7.5%)	0.22±0.07	0
**Trichostrongyloidiasis**	1 (2.5%)	0.45±0.0	0
**Total**	40		3 (7.5%)

**Fig 4:**
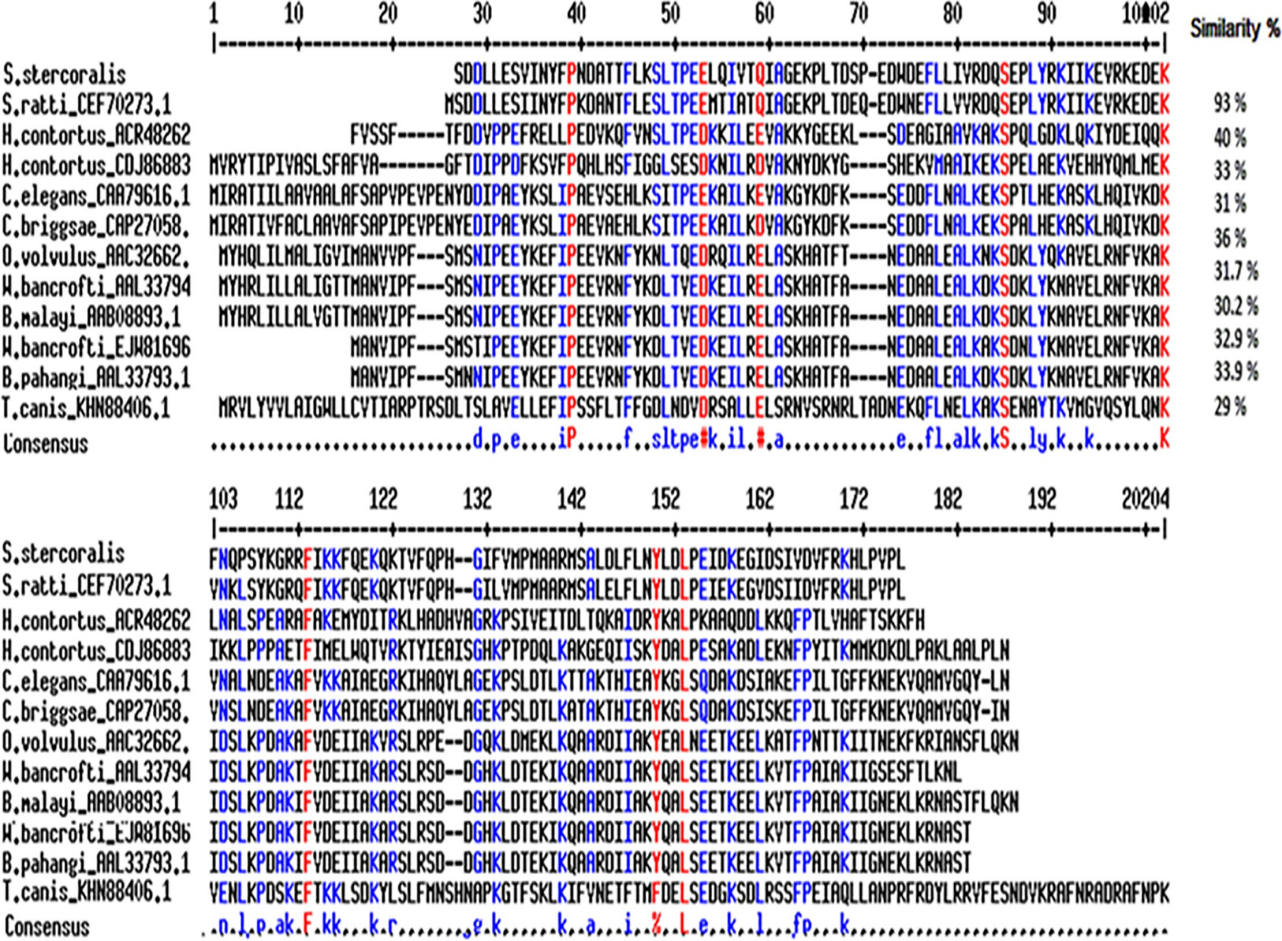
Multiple sequence alignment of fatty acid and retinol-binding protein (FAR) of *S*. *stercoralis*. Fatty acid and retinol-binding protein (FAR) of *S*. *stercoralis* and other nematode including *S*. *ratti (CEF70273*.*1)*, *H*. *contortus (ACR48262*.*1*, *CDJ86883)*, *C*. *elegans (CAA79616*.*1)*, *C*. *briggsae (CAP27058*.*1)*, *O*. *volvulus (AAC32662*.*1)*, *W*. *bancrofti (AAL33794*.*1*, *EJW81696*.*1)*, *B*. *malayi (AAB08893*.*1)*, *B*. *pahangi (AAL33793*.*1)*, *T*. *canis (KHN88406*.*1)* were analyzed by multiple sequence alignment using NCBI protein database, online ClustalW (http://www.clustal.org/clustal2/) and Multalin (http://multalin.toulouse.inra.fr/multalin/) software.

**Fig 5:**
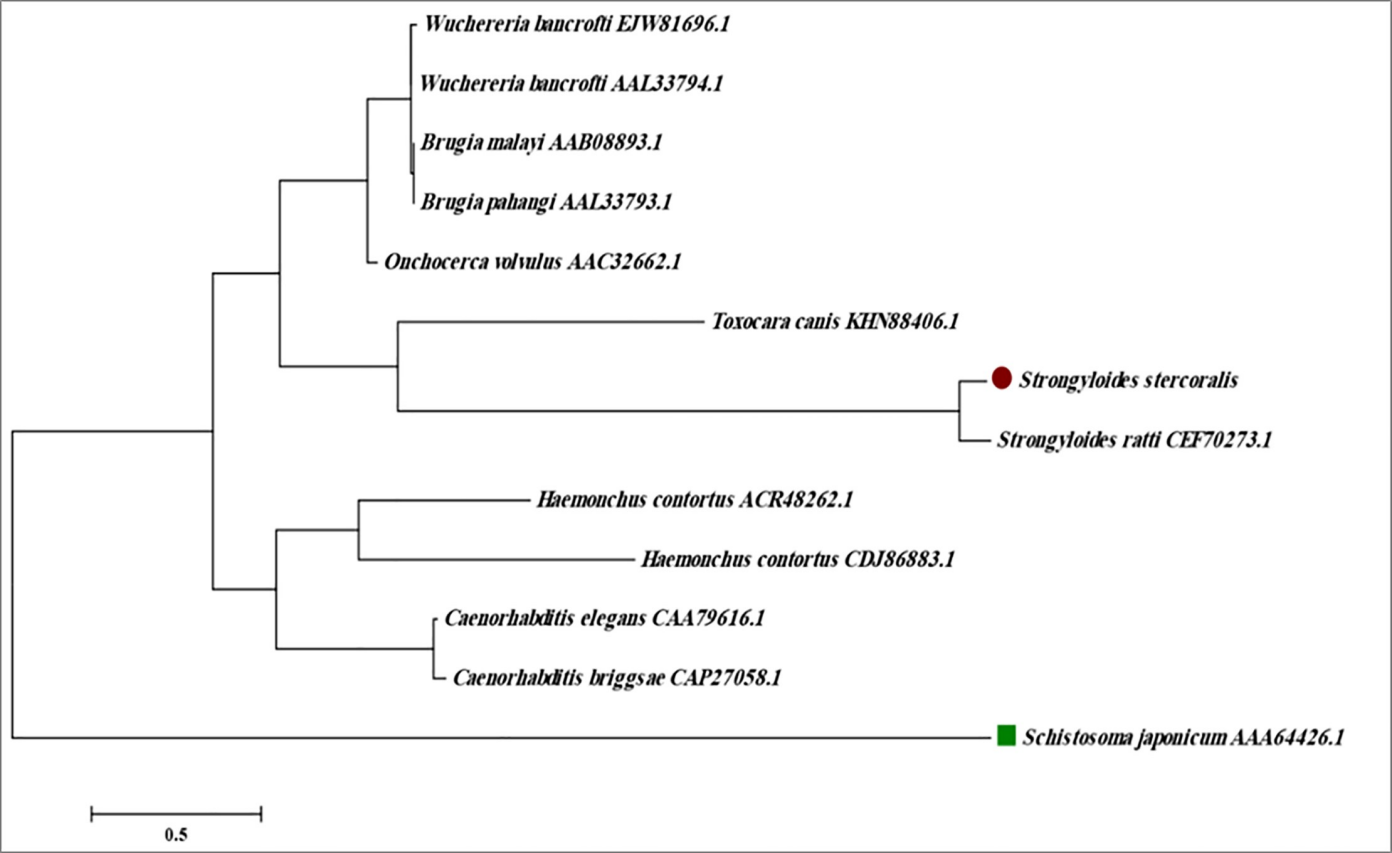
Phylogenetic tree of fatty acid and retinol-binding protein (FAR) of *S*. *stercoralis* with other nematodes. The phylogenetic tree was constructed using amino acids sequences of *S*. *ratti (CEF70273*.*1)*, *H*. *contortus (ACR48262*.*1*, *CDJ86883)*, *C*. *elegans (CAA79616*.*1)*, *C*. *briggsae (CAP27058*.*1)*, *O*. *volvulus (AAC32662*.*1)*, *W*. *bancrofti (AAL33794*.*1*, *EJW81696*.*1)*, *B*. *malayi (AAB08893*.*1)*, *B*. *pahangi (AAL33793*.*1)*, *T*. *canis (KHN88406*.*1)* obtained from NCBI protein database. The Neighbor-joining phylogenetic tree rooted against the FAR protein sequences of the trematode *Schistosoma japonicum* was generated using MEGA 5 software (Arizona State University, Tempe, USA).

Alignment of the deduced FAR amino acid sequence with FAR homologues from other nematodes revealed similarities ranging between 93% (*S*. *ratti*) and 29% (*T*. *canis*) (Fig 4 and S2 File). For construction of the phylogenetic tree, *Schistosoma japonicum* FAR was used as an outgroup (Fig 5).

## Discussion

In this study we cloned and characterized SsFAR protein from the iL3 stage of *S*. *stercoralis*. SsFAR exhibited high similarity (93%) with FAR protein secreted from *S*. *ratti*, and was also similar to a lower degree (29–40%) to FAR proteins of other nematodes.

Immunoblotting with crude *S*. *stercoralis* L3 extract showed that pooled sera from infected patients exhibited considerable reactivity with a 16 kDa protein band. Thus, following the cloning steps, the expression conditions were optimized to prepare an appropriate recombinant antigen to achieve an acceptable sensitivity and specificity in the immunoassay.

The results of immunoblotting confirmed strong reactivity of rSsFAR with sera from strongyloidiasis patients. Thus, rSsFAR was applied for immunodiagnosis of strongyloidiasis by indirect ELISA. The sensitivity and specificity were 100% and 97% respectively, which is commonly regarded to be reliable value for immunological detection of parasitic infections. However, by ELISA, rSsFAR showed cross-reactivity with 2 out of 14 hydatidosis patient sera and also cross-reacted with one out of 9 toxocariasis patient serum samples in ELISA.

Up to now, the potential role of FAR in *S*. *stercoralis* has not been assessed. There is evidence that FAR proteins play a critical role in the development, reproduction and molting of other parasitic and non-parasitic nematodes [41, 48, 49]. In addition, FAR proteins may suppress host defense mechanisms and help *Onchocerca volvulus* to damage the nearby tissues [50]. Our findings are in concordance with previous studies that described FAR proteins as major immunodominant antigens in several infections and point out to their role in the infectivity of some parasites [36].

During the last decades, there was a great effort to produce recombinant immunogenic proteins for application in immunodiagnosis of *S*. *stercoralis*. Many recombinant proteins showed variable sensitivity and specificity, with limited applicability. Up to now, only few proteins were found to be useful in immunodiagnosis of strongyloidiasis. For instance, *S*. *stercoralis* immmunoreactive antigen (SsIR) and a 31-kDa recombinant antigen of its iL3 form (NIE) revealed high reliability. Ravi et al. demonstrated that coating of ELISA plates with NIE resulted in seropositivity in 78.5% of the patients and 6.6% of the controls [51]. Recently, application of luciferase immunoprecipitation system (NIE-LIPS) improved the sensitivity and specificity of NIE-specific ELISA and gained good sensitivity (75–98%) and specificity (94–100%) [20, 51–53]. Mounsey et al. utilized the recombinant NIE antigen for analysis of dried blood spots and showed that this method has a good diagnostic performance with 85.7% sensitivity and 88.9% specificity [54]. This method also elucidated comparable sensitivity and specificity to routine NIE-ELISA, thus, it seems that the collection of dried blood spots may be a useful approach for seroprevalence screening of strongyloidiasis [54]. Rascoe et al. employed NIE antigen in a standard ELISA and in a fluorescent bead-based assay (Luminex) to detect *S*. *stercoralis*-specific IgG4. The sensitivity and specificity of this antigen in ELISA was 95% and 93%, respectively; while, the sensitivity and specificity for Luminex assay was 93% and 95%, respectively [55].

Arifin et al. succeeded in cloning and identification of Ss-1a protein from a *S*. *stercoralis* cDNA library and demonstrated its potential role in diagnosis of strongyloidiasis. Moreover, they concluded that Ss-1a has a high similarity with immunoglobulin-binding protein 1 of *S*. *ratti* and reported 96% sensitivity and 93% specificity for this novel recombinant protein [56]. More recently, we applied recombinant 14-3-3 in ELISA systems to detect anti-*S*. *stercoralis* IgG antibodies and found that the sensitivities of the 14-3-3-ELISA assays was 96% [57]. A comparative analysis should be undertaken to evaluate the performance of rSsFAR-based ELISA in comparison to other recombinant antigens. In this study, we fund that rSs-FAR is an excellent recombinant protein for serodiagnosis of human strongyloidiasis. According to our findings, rSs-FAR protein has a relatively higher sensitivity and specificity than other proteins used to detect the IgG titer in the patients sera.

Furthermore, it seems that rSs-FAR may be also applied for immunoprophylactic purposes, too; however, its effectiveness as a recombinant immunogen should be further investigated.

In this work, we encountered some problems such as low accessibility to enough number of serum samples from other parasitic diseases for inclusion in ELISA; thus, we could not broadly investigate the cross-reactivity of *S*. *stercoralis* antigens with other parasites which demonstrate similar symptoms. Hence, further evaluation of additional serum samples may provide more reliable interpretation of the obtained results.

## Conclusions

In conclusion, we cloned the cDNA coding for the fatty acid and retinol-binding protein of *S*. *stercoralis* (SsFAR), obtained the full-length sequence, and expressed it as a recombinant antigen (rSsFAR) in bacteria. We found that rSsFAR protein could be considered as an alternative diagnostic tool in ELISA for the detection of *S*. *stercoralis* infections, with a sensitivity and specificity of 100% and 97%, respectively. Finally, since SsFAR is an immunodominant antigen, in addition to its possible applications in immunodiagnosis, it could be used in vaccinology studies against *S*. *stercoralis* infections.

## Supporting information

S1 File Alignment of oligonucleotides. **Alignment of oligonucleotide sequences of Fatty acid and retinol-binding protein (FAR).** Oligonucleotide sequences from Strongyloides and other nematodes which were used for alignment and determination of their homology.
(TXT)

S2 File Alignment of proteins. **Alignment of protein sequences of Fatty acid and retinol-binding protein (FAR).** Protein sequences from Strongyloides species and other nematodes which were used for alignment and determination of their homology.
(TXT)

S3 File FAR-ROC Test. **Receiver-operating characteristic (ROC) curves data for Fatty acid and retinol-binding protein (FAR).** Graph-Pad Prism software were applied to plot ROC curve using ELISA optical density (OD) results of strongyloidiasis patients and healthy controls.
(PZFX)

S4 File Anova Test-Prism FAR. **Anova test for cross-reactivity of Fatty acid and retinol-binding protein (FAR).** Graph-Pad Prism software were applied to evaluate the ELISA results and determine the cross-reactivity of *Strongyloides stercoralis* recombinant FAR protein with other parasitic diseases.
(PZFX)
